# Designing a Mobile Health Solution to Facilitate the Transition from NICU to Home: A Qualitative Study

**DOI:** 10.3390/children9020260

**Published:** 2022-02-15

**Authors:** Ashwini Lakshmanan, Isabel Sunshine, Sam Calvetti, Juan Espinoza, Sofia Santoro, Saloni Butala, Madison House, Michele Kipke

**Affiliations:** 1 Division of Neonatal Medicine, Fetal and Neonatal Medicine Institute, Children’s Hospital Los Angeles, Keck School of Medicine, University of Southern California, Los Angeles, CA 90027, USA; isunshin@usc.edu (I.S.); sesantor@usc.edu (S.S.); sabutala@chla.usc.edu (S.B.); mhouse@chla.usc.edu (M.H.); 2 Leonard D. Schaeffer Center for Health Policy and Economics, University of Southern California, Los Angeles, CA 90089, USA; 3 Department of Population and Public Health Sciences, Keck School of Medicine, University of Southern California, Los Angeles, CA 90033, USA; 4 Division of Research on Children, Youth and Families, Children’s Hospital, Keck School of Medicine, University of Southern California, Los Angeles, CA 90027, USA; scalvetti@chla.usc.edu (S.C.); mkipke@chla.usc.edu (M.K.); 5 Division of General Pediatrics, Children’s Hospital Los Angeles, Keck School of Medicine, University of Southern California, Los Angeles, CA 90027, USA; jespinoza@chla.usc.edu; 6 Saban Research Institute, Children’s Hospital Los Angeles, Keck School of Medicine, University of Southern California, Los Angeles, CA 90027, USA

**Keywords:** transition, NICU, mobile health solution, application, discharge

## Abstract

There is limited information about caregiver and provider perspectives regarding the design of a mobile health solution to facilitate the transition from the neonatal intensive care unit (NICU) to home. Focus groups were conducted with English- or Spanish-speaking families enrolled in an urban high-risk infant follow-up clinic and with their care providers. We generated salient themes using an inductive thematic analysis. Twenty-two participants completed the study. Among caregivers, the infant’s median gestational age (IQR) was 29 (23, 34) weeks and 63% were Hispanic. Among the providers, 55% had practiced for more than 10 years and 18% were bilingual. Key stakeholder (family and provider) priorities for designing a mobile health solution were organized into eight domains, i.e., implementation ideas around user interface and timing, providing path planning and information, increasing support, improving engagement with providers and services, mitigating barriers to care after discharge and strengthening parenting role and confidence. The results from this study suggest that families and healthcare providers prioritize path planning, information and support as the pillars for designing an effective NICU-to-home transition mobile health application. Implications for product development include family empowerment, being a credible source of information and creating a resource for caregiver support and mental health.

## 1. Introduction

The transition to home for infants hospitalized in the neonatal intensive care unit (NICU) poses unique challenges, in terms of communication between parents and providers, hospital discharge processes and the psychosocial and emotional status of caregivers [1]. This critical handoff in the care of medically complex infants has been investigated with an increased emphasis on parental and caregiver perspectives [2,3,4]. There has also been increased attention to disparities in social determinants of health that exist for families as they embark on this challenging transition of care [4,5,6]. The American Academy of Pediatrics’ updated policy statement entitled “Hospital Discharge of the High-Risk Neonate” identified parental education as one of the six critical components for discharge planning of this patient population [7], further emphasizing the irreplaceable role that caregiver confidence and competence plays in this process. Caregiver preparation is essential for ensuring this process proceeds smoothly and must include consideration of parental roles, emotional support, adequate knowledge and training for appropriate management of medically complex infant care [2]. Hospital readmissions among NICU graduates remain more frequent than their full-term counterparts, with non-White race/ethnicity, primary language other than English and lower SES all contributing to increased rates of readmissions [8]. Moreover, minority and low-income families have lower rates of service intensity and higher rates of attrition from prescribed early intervention (EI) services [9]. Although the transition of care from the NICU to home is not an understudied area, there remains a need for novel solutions to the many unique challenges parents and providers face.

When considering potential improvements for the NICU-to-home transition, web-based and mobile applications (apps) remain a largely underutilized potential solution. The use of websites and mobile apps for health management and education have risen in popularity, especially with the increased need for healthcare delivery through telemedicine in the past few years [10]. In fact, many parents already utilize the internet and mobile apps to supplement and support their role in development and education as new parents [11,12]. Unfortunately, many of the currently available internet websites and mobile apps may be unreliable in terms of the quality of information they provide [13,14]. In a systematic app review performed by Richardson et al. in 2019, less than half of the available apps targeting NICU parents were considered acceptable educational material [14]. Mobile applications for facilitating the transition from the NICU to home have already proven to be a promising strategy for improving parental preparation for discharge [15], yet the availability of apps specifically created with consideration for the unique desires and needs of caregivers and providers at safety-net centers remains limited.

This study was designed to elucidate caregiver and provider perspectives on the design of a mobile health application for facilitating the transition from the NICU to home. Our aim is to identify general recommendations and specific priorities of caregivers and providers in terms of the content, design, information and support that such an app might provide. This focus on user-centered design could help to address existing concerns regarding the quality, reliability and utility of mobile apps dedicated to facilitating a successful transition from the NICU to home for high-risk infants. Through the qualitative analysis of focus group interviews with families enrolled in an urban high-risk infant follow-up clinic and with providers who care for them, we identified eight domains for family and provider priorities for the future development of a mobile health application, namely, implementation ideas around user interface and timing, providing path planning and information, increasing support, improving engagement with providers and services, mitigating barriers to care after discharge and strengthening parenting role and confidence.

## 2. Materials and Methods

### 2.1. Research Team and Reflexivity

Focus groups were conducted by the author (A.L.), a neonatologist and research staff (S.C.) with experience in qualitative methods. Both the participants and research team had knowledge of the infants’ diagnoses and history. Participants were aware of the research objectives of the study.

### 2.2. Study Design and Framework

This was a qualitative prospective study that leveraged an inductive thematic analysis as our methodologic framework. We opted to conduct focus groups to make possible repartee among participants to generate ideas.

### 2.3. Participant Selection and Setting

Focus groups were conducted in 2018. The participants were recruited using purposive, typical case sampling. The participants were approached using face-to-face or telephone recruitment. Caregiver participants were recruited from a high-risk infant follow-up clinic at a quaternary urban safety-net children’s hospital that serves a primarily Medicaid-insured population. This clinic provided multidisciplinary follow-ups for infants with gestational age of <32 completed weeks or birth weight < 1500 g, or infants who received extracorporeal membrane oxygenation or therapeutic hypothermia or had other problems that could result in neurologic abnormality. Provider participants were key stakeholders who cared for infants in the NICU, at the high-risk infant follow-up clinic, or in general pediatrics. They were approached using face-to-face or email recruitment.

We included English- or Spanish-speaking parents if enrolled in Medicaid for infants with complex health care needs. Families were approached at the initial visit after NICU discharge or at one of the three standard visits recommended by the California Children’s Services High Risk Infant Follow Up (HRIF) program (4–8 months, 12–16 months and 18–36 months of corrected age). We included a range of enrollment times to understand the spectrum of issues in regards to barriers and facilitators during the transition to home. The focus groups were conducted by telephone and the participants (caregivers and providers) were given a monetary incentive upon completion of the focus group. If the focus group was conducted in Spanish, interpretation services were used. The Children’s Hospital Los Angeles (CHLA) human subjects protection program approved the study protocol and all subjects provided informed written consent. We finished recruitment once thematic saturation was reached.

### 2.4. Development of Interview Guide

A literature review and key stakeholder (informant) interviews with families, neonatologists, developmental pediatricians, clinical care coordinators and nurses informed the interview guide. During data collection, we refined the guide (Appendix A, Table A1) in an iterative fashion and pilot-tested it prior to administration. Field notes were collected during all interviews and focus groups.

### 2.5. Data Collection

After recruitment, we scheduled focus groups. The focus groups were conducted as providers only and as families only. There were 2–4 participants in the family focus groups and 6–10 participants in the provider groups. The provider focus groups were conducted in person and the family focus groups were conducted by telephone due to participant schedules. We opted for telephone-based focus groups for families since many families opted for telephone-based (rather than video-based) focus groups. Since the data were collected pre-pandemic, many families were uncomfortable with video-based services. They were recorded on a digital recorder and then transcribed by a third-party service. Focus groups were 60–90 min. When participants finished their focus group, they received a gift card. Caregiver demographic data and data about the infant’s health were extracted from the medical record.

### 2.6. Data Analysis

Transcripts were imported into Dedoose Version 8.3.35, web application for managing, analyzing and presenting qualitative and mixed method research data (2020) (Los Angeles, CA; SocioCultural Research Consultants, LLC; www.dedoose.com, (accessed on 10 February 2022) [16]). We used an inductive thematic analysis approach, an iterative process of coding to identify the patterns among concepts. Two coders (S.B. and M.H.) shared initial coding duties and a third coder (S.S.) double-coded interviews to increase validity. The codebook was modified as necessary and discrepancies were resolved through consensus; we kept an audit trail. Our approach was to develop a set of codes from repeating themes in the data and to conduct co-coding until we had consensus. The ultimate codebook contained 25 codes. In total, 947 unique excerpts were coded between 1 and 8 codes each.

## 3. Results

### 3.1. Demographics

As shown in Table 1, there was a total of 22 participants, 11 caregivers and 11 providers. The majority of infants were Hispanic (63%) and the median (IQR) gestational age was 29 (23, 34) weeks. All infants were categorized as having complex chronic disease (C-CD) using the Pediatric Medical Complexity Algorithm [17], with nearly half having sensory issues and global developmental delays and the majority (73%) receiving early intervention, including speech, occupational/physical and visual therapy. Regarding caregiver demographics, all participants were on Medicaid/California Children’s Services, the majority categorized English as being their first language (81%) and more than half (63%) lived in a service planning area with a 4th quartile economic hardship index. In terms of providers, the most common were neonatologists and nurses, 37% and 27%, respectively; almost all were female (90%) and more than half had more than 10 years of clinical experience (55%).

### 3.2. Conceptual Model

**Figure 1:**
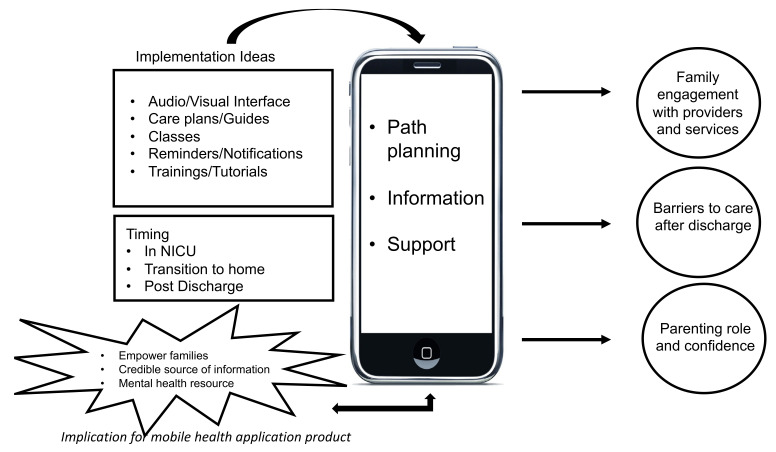
Conceptual model illustrating domains regarding parents’ and providers’ priorities for mobile health application for the hospital-to-home transition.

The conceptual model illustrated in Figure 1 highlights the following eight key domains that encompass the identified priorities of parents and providers during the hospital-to-home transition: ideas for content formats and user interface (1), timing of events around discharge (in the NICU, around transition and post-discharge care) (2), path planning (3), provision of information (4), accessing support (5), engagement with providers (6), barriers to care after discharge (7), parenting role and confidence (8).

### 3.3. Domain 1: Ideas for Content Formats and User Interface

The core themes represented in this domain included providing audio/visual resources, care plans/guides, classes around discharge/transition issues, reminders and notifications, and tutorials (Table 2). Audio and visual resources were desired as they would not only be more inclusive for parents who may be illiterate or non-native speakers but would also assist parents in digesting dense medical information and help “build confidence”. In terms of care plans and guides, parents expressed their struggles with “keeping track of everything” and how “printouts” or “simple manuals” would help them navigate caring for their child independently. Providers agreed that “guides” should be provided and suggested that they would aid in guiding families through the weeks following discharge. Classes were overwhelmingly requested by parents (*n* = number of participants) (27) compared to providers (4). Even when already receiving other resources, parents felt that classes were essential in providing information and skills to help care for their infant and would like to see a greater number of accessible classes. One mother stated the following: “the more classes I went to, the more knowledge I got from other people, and resources, the better I became, and the easier it became”. Both notifications and tutorials were positively received, with many providers and parents alike expressing how both would be beneficial in receiving critical information.

### 3.4. Domain 2: Timing of Events around Discharge

This domain addressed the theme of timing of events around discharge for patients in the NICU, during the transition period and post-discharge (Table 2). Parents expressed concerns about independently caring for their child at home for the first time. A first-time mom voiced her concerns and fears regarding leaving the hospital with her special needs child and wondered “if the baby is going to live”. Providers provided further insights about how to prepare and guide parents through each stage and highlighted the “setting up of educating the family on what is expected of them … what kind of tasks they need to learn as well as the timeline” of those tasks is crucial in helping parents gain a sense of self-efficacy. As the transition from the NICU to post-discharge care can be difficult, providers emphasized offering parents discharge plans “so that they can focus their attention to that next step”.

### 3.5. Domain 3: Path Planning

Themes in this domain included providing a discharge summary, patient navigation assistance with parent navigators, coordination with the medical home (team-based coordinated care) and facilitating appointment scheduling/referrals to specialists (Table 3). Providing a discharge summary was exclusively commented on by providers, many of which advocated for the distribution of updated discharge summaries to parents. On the other hand, parent navigation assistance was exclusively commented on by parents, who expressed a need for more parent mentors and navigators to help guide them through post-discharge challenges, such as insurance or scheduling. Parents expressed a general lack of awareness with the concept of medical home, while providers provided more insight about the benefits of medical home. Providers described medical home coordination as having the ability to address and solve parent anxiety, as it can provide information about medical equipment, insurance coverage, medications and contacting providers. Appointment scheduling and referrals were also identified as being barriers to care. Due to the high demand of medically complex babies, parents recounted their struggles with balancing appointments with other personal commitments.

### 3.6. Domain 4: Provision of Information

Themes from this domain were split into three main requested content areas, i.e., accessing and using medications/durable medical equipment, accessing community-based services and accessing mental health services (Table 3). Administering medications and using durable medical equipment, where to obtain them and how to use them without professional assistance were of great concern to many parents. Despite receiving pre-discharge training and instructions, parents still expressed apprehension; one parent stated the following: “And if you accidentally miss this because you’re so worn out and tired, then you’re gonna mess up everything … it’s nerve-racking knowing that all of this is soon gonna be your, your issue, your things to deal with”. Another requested content area was community-based services, with possible links and connections to food vouchers, diaper certificates, or even regularly scheduled family support groups. Lastly, parents and providers agreed that mental health services, unlike other services, appeared to be very inaccessible to parents.

### 3.7. Domain 5: Accessing Support

Four primary types of support emerged from this domain, including family support, peer support, religious support and support groups (Table 3). Despite encountering difficulties post-discharge, many parents found support from their families, whether that was their parents, siblings, spouses, or their child’s godparents. Family support came in the form of caring for other children, assisting with medical supplies and being a source of comfort during times of stress and overwhelming emotions. Moreover, parents found solace in interacting with and receiving advice from peers who had undergone similar experiences with their medically complex children. Religious support was exclusively discussed by parents, who explained that they found support through religion by attending church, speaking with religious figures, or visiting prayer centers. Parents and providers expressed that support groups, in both virtual and face-to-face settings, were certainly of importance. Support groups were described as important spaces for parents to create a network of resources, combat feelings of loneliness or isolation, as well as receiving advice from other parents that may be more experienced.

### 3.8. Domain 6: Engagement with Providers

This domain had two primary themes, which included communication with pediatricians, as well as communication and importance of clinical care coordinators (Table 4). For parents, clinical care coordinators and other providers, an intense amount of effort was needed to call various pediatricians to check for availability, retrieve patient information, or provide pediatricians with updates. One provider described their experiences of having to make repeated calls to ask, e.g., “You know, this baby with X, Y, Z, can you, do you have the resources to take care of it in your clinic?” and often receiving pushback. Similarly, a parent described their miscommunication with a provider and how that made them feel “inadvertently blamed” for their child not improving medically. As outlined by both parents and providers, it was clear that parents must advocate for their child when it comes to accessing pediatricians and specialists. Parents expressed appreciation for clinical care coordinators; however, it was noted that such coordinators were, at times, inaccessible or they were not previously aware of them. One parent described clinical care coordinators as being “amazingly helpful”; however, they were not aware of “how much they could help”, since they were not initially given their contact information.

### 3.9. Domain 7: Barriers to Care after Discharge

Within this domain, three major barriers to care following discharge were identified, that is, health literacy, language/cultural barriers and transportation/housing (Table 4). Complex language and medical jargon were highlighted as a major barrier to understanding the child’s needs and seeking care for such needs, particularly for those who may be at a lower reading level, or those whose primary language is not spoken by their provider. Language and cultural barriers were solely touched upon by providers. Language barriers not only make communication difficult between patient and provider, but may also prevent that patient from seeking other resources outside the hospital. It was highlighted by one provider that “having a support group that you know is non-judgmental” could possibly address some cultural differences and barriers to obtaining support and clinical care. As expressed by patients and providers, transportation was a major area for improvement, as many patients may not have feasible ways of attending appointments, particularly for those who only walk or use public transportation.

### 3.10. Domain 8: Parenting Role and Confidence

This domain highlights parenting confidence and parenting roles (Table 4). The main theme of this section was how a parent’s confidence and the lack thereof played a role in how they provided for their infant. Among parents, there seemed to be difficulties relating to peers who did not have a premature baby, with one parent stating they were “just scared because they had never seen a premature baby”. Providers also highlighted confidence as important, e.g., “It is the same lack of confidence that, that families in the NICU feel except for the fact that this child is more likely to have a big setback with their lack of confidence. And that is what drives the fear and the anxiety”. Despite parent unfamiliarity with caring for high-risk babies, another provider noted that there are ways to build that confidence, such as remaining bedside, which helps the parent gain awareness of their baby and create closer bonds and, in turn, “builds their confidence”, since “they know that they can provide for their baby” despite their lack of experience.

### 3.11. Implications for Product Development

**Figure 2:**
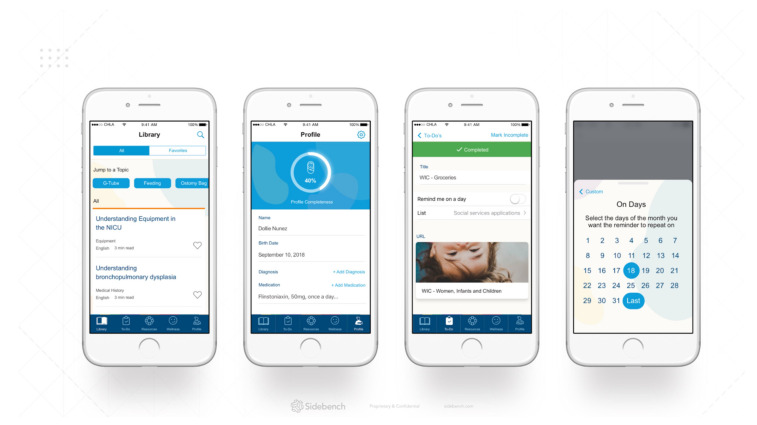
Template design of app.

The implications for product development include focusing on family empowerment, being a credible source of information and creating a resource for caregiver support and mental health. The wireframe design of a mobile health solution that meets these needs is shown in Figure 2.

## 4. Discussion

Caregiver and provider priorities for designing a mobile health solution during the transition from the NICU to home included the following domains: implementation ideas around user interface and timing, providing path planning and information, increasing support, improving engagement with providers and services, mitigating barriers to care after discharge and strengthening parenting role and confidence. Implications for product development include family empowerment, being a credible source of information and creating a resource for caregiver support and mental health. The data collected in this study were particularly unique as we focused on participants in a safety-net setting.

Families and healthcare provider participants prioritized path planning, information and support as pillars for designing an effective mobile app. Both parties described a variety of organizational processes that must occur to achieve a smooth transition from the NICU to home, including the provision of clear discharge instructions, coordination with the medical home and appointment scheduling with general pediatricians and specialists. These findings are consistent with prior studies which described parent perspectives on the transition to home with their infants. Of note, families emphasized the importance of patient navigation and providers focused on discharge summaries. Berman et al. found that communication was a key challenge for parents when they self-assessed their preparedness for discharge [2]. Enlow et al. also described how communication with healthcare staff was of greater concern to caregivers than was their access to concrete resources such as transportation [4]. Provision of clear discharge summaries and patient navigators within the proposed mobile application are both opportunities for improvement in communication and could streamline and consolidate the frequently daunting amount of information that caregivers receive during the discharge process. Multiple studies of caregiver perspectives on this transition have also identified self-efficacy as a key component for parental readiness [2,3]. Desai et al. described how different forms of support and communication improved caregiver perception of self-efficacy, including clear discharge planning and provider support following discharge, which were also identified by our study participants as desirable components of path planning within a mobile app [3]. Feasibility of a shared care plan has already been proposed and studied by Desai et al. [18], where the researchers studied parent and provider perspectives on the creation of a cloud-based shared care plan for children with medical complexity. Based on the findings in our study, we believe that the creation of a smartphone application that includes multiple path planning features would fulfill the desires of parents to have an accessible, user-centered and collaborative way to engage with their child’s providers and health information. While families and providers had different priorities in the process, they both focused on the importance of path planning.

Families and providers generally desired a mobile app with more information for caregivers, especially surrounding accessing and using medications and durable medical equipment, connecting to community-based services and accessing mental health services. Hebballi et al. previously noted that parents already turn to their smartphones and social media platforms to supplement their knowledge of their infant’s medical care and to keep track of their child’s appointments, medications and other medical tasks [1]. Thus, our study participants’ desire to utilize a mobile application for accessing and using medications and for locating durable medical equipment aligns with the current understanding of parental smartphone usage to assist in caring for their infants after NICU discharge. In regards to accessing community-based services, the providers in our study identified completion of referral to early intervention as an important element of information provision for parents which was consistent with previous findings [19]. In their investigation of possible internet-based interventions for ensuring prompt connection to early intervention (EI) services, Baggett et al. found that the “most common reason for mothers and their infants to fall off the path toward intervention was that they could not be contacted after leaving the NICU” [20]. Parent and provider preferences from our study suggest that, by providing information and referral updates for EI services through a mobile app, the concern for missed referrals in this vulnerable patient population could be improved upon. Our participants’ desire for an app which contains information on accessing mental health services is consistent with a McGowan et al.’s study, which previously demonstrated that mothers with a history of mental health disorders (MHDs) perceived themselves as less ready for their infant’s discharge than did mothers without a history of MHDs [21]. This study and our participant’s mobile app preferences emphasizes the importance of connecting caregivers with appropriate mental health services prior to discharge and during the transition to home care of their infant. We also identified that families were focused on more technical issues such as durable medical equipment, mental health etc., while providers were more focused on transitions of care (to community-based services, etc.).

The desire for a variety of support systems including family, peer and religious affinity support groups was another common theme amongst both family and provider perspectives on important components of a mobile health app for supporting the transition from the NICU to home. Franck et al. previously reported that, in interviewing parents about their NICU discharge and transition to home experiences, “all parents needed support or someone to talk to” when the “magnitude of what they had been through was realized” [22] and multiple other studies have more specifically demonstrated the importance of peer-to-peer support for caregivers [23]. Parents of infants who have been hospitalized in the NICU also experienced a unique anxiety surrounding their child’s perceived fragility, which gathering in and speaking with a community of peers may help to normalize [24]. Furthermore, the different kinds of support groups or communities that families and providers proposed as potentially helpful components of a mobile app is consistent with previous studies of the variety of parental experience when making the transition with their infant from the NICU to home. This diversity of experience and of desired support groups is reflected by differences in infant medical complexity, caregiver socioeconomic status and maternal immigrant status, for example [3,24,25].

Designing a mobile health application for caregivers of infants hospitalized in the NICU and to support them in the transition of their child from the NICU to home is a novel concept that has not been previously studied. In their randomized control trial of a smartphone application to support parents of premature infants during this transition, Garfield et al. found that, with increased use of their NICU-2-home smartphone app, parents reported increased sense of competence and discharge preparedness [15]. Our study is uniquely positioned to provide a conceptual model for the development of a mobile app tailored to the needs of parents and to the recommendations and expertise of healthcare providers from the NICU. While our results demonstrate that path planning, information and support are all desired areas of content for a mobile app, our findings in terms of parent and provider implementation ideas can also serve as a helpful framework for future app development. Overall, an application developed based on this model could empower families, serve as a credible source of information and be an important source of mental health resources and services. It is also important to consider access to stable data plans and internet when designing products [26].

There are multiple implications for practice. We suggest that providers consider taking the following actions:

(1) Consider the access to technology that families might have (type of smart device, carrier, etc.);
(2) Integrate educational and path-planning resources into discharge planning using web-based tools such as a mobile health applications or websites;
(3) Consider virtual support groups or structures to support families during the transition to home.

In terms of limitations of this study, we purposively sampled patients from a safety-net center because we wanted to learn more about their perspectives on a mobile health transition application. Therefore, their opinions may not reflect those with higher socioeconomic status. Moreover, we only sampled providers from a single center. Our findings may not reflect all providers; however, we did take great care to recruit a variety of key stakeholders caring for high-risk infants and their families. Additionally, telephone interviews may have limited the ability of participants to build trust with the research team and they may not have been as forthright with their responses. Subsequent research in other comparable centers might yield additional insights about mobile health product development.

## 5. Conclusions

The results from this study suggest that families and healthcare providers prioritize path planning, information and support as the pillars for designing an effective NICU-to-home transition mobile health application. We believe that, by prioritizing the perspectives of both parents and providers, a mobile application could mitigate existing barriers to care after discharge and enhance family engagement with providers and services.

## Figures and Tables

**Table 1:** Participant demographics (*n* = 22).

	N (%)	Median (IQR)
Infant and Parent Demographics (*n* = 11)		
Infant demographics		
Race/Ethnicity		
Hispanic	7 (63)	
Black non-Hispanic	2 (19)	
White non-Hispanic	1 (9)	
Other (Native American, Mixed)	1 (9)	
Gestational age (weeks), median (IQR), if preterm		29 (23, 34)
Birth weight (grams), median (IQR)		1365 (969, 2800)
Neonatal diagnoses		
Bronchopulmonary dysplasia	5 (45)	
Retinopathy of prematurity requiring intervention	3 (27)	
Necrotizing enterocolitis (surgical)	1 (9)	
Intraventricular hemorrhage (Grade III/IV)	2 (19)	
Infants with medical complexity (post-discharge) ^a^		
Complex Chronic Disease (C-CD)	11 (100)	
Non-Complex Chronic Disease (NC-CD)		
Non-Complex Non-Chronic Disease (NC-NCD)		
Infants requiring prescription medications after discharge	4 (36)	
Infants requiring durable medical equipment after discharge	4 (36)	
Oxygen	3 (27)	
Feeding tube (gastrostomy/jejunostomy)	4 (36)	
Diagnosis of developmental issue after discharge (may have more than 1)		
Language delay	1 (9)	
Autism risk	1 (9)	
Sensory issue	5 (46)	
Global developmental delay	5 (46)	
Cerebral palsy	3 (27)	
Early intervention services received	8 (73)	
Speech therapy	3 (27)	
Occupational/Physical therapy	5 (45)	
Vision	1 (9)	
**Maternal demographics**		
Primary Language		
English	9 (81)	
Spanish	2 (19)	
Neighborhood equity ^b^		
Home zip code in service planning area (SPA) with 4th quartile economic hardship index	7 (63)	
Medicaid/California Children’s Services	11 (100)	
Distance to HRIF clinic from participants’ home, in miles, median (IQR)		12.2 (5.8, 41.8)
Distance to early intervention program from participants’ home, in miles, median (IQR)		4.4 (1.7, 15.6)
**Provider Demographics (*n* = 11)**		
Provider type		
Neonatology (physician)	4 (37)	
Clinical care coordinator/Case Manager	1 (9)	
Nursing	3 (27)	
Social Work	1 (9)	
Developmental behavioral specialist	1 (9)	
General pediatrician	1 (9)	
Years of practice		
<5 years	2 (18)	
5–10 years	3 (27)	
>10 years	6 (55)	
Gender (female)	10 (90)	
Participate in transition planning/discharge planning committees	4 (37)	
Bilingual provider (English and Spanish)	2 (18)	

^a^ Defined by Pediatric Medical Complexity Algorithm (version 3.0) (measurement tool accessed at: https://www.ahrq.gov/sites/default/files/wysiwyg/pqmp/measures/chronic/chipra-141-fullreport.pdf, (accessed on 10 February 2022)); ^b^ Service planning area 6, 7, 8 (4th and highest quartile of economic hardship index).

**Table 2:** Themes and illustrative quotations: implementation ideas (content formats, user interface and timing).

	N	Patient		Provider	
Domain/Theme		N	Illustrative Quotations	N	Illustrative Quotations
Domain 1: Ideas for content formats and user interface					
(a) Provide audio/visual resources	29	17	“I’m a very visual person, I [want] to click right to that video, and after I’m done with it, I can also read an article” “Definitions, medical terminology, like little sketches or PowerPoint presentations or some type of thing where it shows you direct step by step on how to deal with certain procedures afterwards, like even if they’re just more like redo your information on how to, how to place a bag, or how to, correctly change out a G tube, whatever, or how to do a trach, anything, just like step by step, and, and like watching somebody actually do it. Yeah that would be awesome, and clicking on certain words for medical terminology, you get a better definition and understanding of it. Being able to talk at a medical professional with a click of a button if you have any other questions, that’s available to you 24/7.”	12	“[There is] an online, electronic, learning tool called the GetWellNetwork. The NICU doesn’t have it, and that’s a big setback for us. But that builds confidence in that they can watch a video, audiovisual, before they practice. Every nurse has a different style in how they teach. Some nurses will schedule a teaching, some nurses will turn around and say, hey, I’m about to do this, step right up. Not every family is comfortable doing something for the very first time on their child with the implications of risks and, and that kind of thing without having watched it first, maybe rewind, watch it again.” “We had an illiterate mom, so we allowed her to record [us] teaching her, so she would have that as her teaching because she would not be able to read the discharge instructions. And so, that’s a special circumstance and it wasn’t a fancy device, but it was us using technology in a way. We’d love to use it in the future, to teach.”
(b) Provide care plan/guides	12	9	“I’d love it if there was something like that in the NICU, [because] he was on so many more drugs then, and I had such a hard time keeping track of everything. I think for me having it like written down or even if they could kind of give you like printouts… it was kind of this blur for me.” “I would love it if there were some way to some sort of a simple like manual for our [oxygen] machine too… if the oxygen saturation is below like or hits 98% versus 100 then it starts beeping. I’m going, how do I stop this incessant beeping?”	3	“When when I was thinking through these things, we should consider providing a guide like there is the ‘100 Days of Autism.’ So it’s like day one, what are you supposed to be thinking of, day two. Like it’s a day by day thing that guides some, a parent through a first diagnosis of autism. And so something, like you’re saying, like a timeline or something with the NICU, like these are during the first week when you’re out of the NICU, consider these things. During the second week, consider these things, or the first month.”
(c) Consider providing classes around discharge/transition issues	31	27	“Being a first-time mom with a child with a disability, they gave me a lot of resources, but even with the resources… Like, seriously even though I took the CPR classes and everything I still panicked a couple of times. But eventually the older she got the more better I got. The more classes I went to, the more knowledge I got from other people, and resources, the better I became, and the easier it became.” “The parenting hour that they would have… I liked those so much because they would have different subjects that they would touch on in each class. And they would really give you some very vital information that you needed. They would even have other parents come in and talk to you. They would have different nurses come in and talk to the group. And it was really, really very helpful. I just wish, I honest to god wish that there was more of those classes every week, instead of just like one or two.”	4	“Wouldn’t it be great if a family who knew how to put a G-tube in could, could rotate around and could do classes or… [another provider} got a grant from the Regional Center to help bring in a family navigator to help families navigate the Regional Center with classes.”
(d) Reminders and notifications	18	10	“I think it’s very useful to remind you of any appointments or anything that you have to do at the clinic or hospital.”	8	“Maybe direct links, if you can attach ‘em on, instead of them having to go through, five different links in one website, have the direct form with reminders.” “Connection to updates from the medical team and therapy services. As in, they might get a message, ‘did you forget your refill?’ Or they might get a message ‘Follow up with surgery in two weeks,’ like a bulletin board I suppose.”
(e) Tutorials	46	29	“Actually, I like how you guys have tummy time tutorials… parents hours are absolutely amazing.” “Do they have an option for how to switch out the G-tube, like, step-by-step instructions?”	17	“I would imagine that [tutorials] would also help standardize discharge teaching too because we have wildly different teaching styles.” “Because you may not know how to fill out that information… And then there might even be a way to say if you don’t have this number, like this is how you would obtain it and have like side links off that, so that you don’t get stuck on one component of it and not be able to complete the rest of the form.”
Domain 2: Timing of events around discharge (In the NICU, around transition and post-discharge care)	40	14	“Well my fears when I left were being a first-time mom you’re kind of scared, especially with special needs children, because you wonder if she’s [the baby] is going to live to say am I doing it right...guiding first time moms is important.” “She has a lot of therapy. So, just trying to like, have a good schedule for her and figuring out when is what… somehow, someway, we’ve been pulling through good.”	26	“Like in our discharge [plan], I think we have different, time intervals, for what’s relevant in that time. So dividing it from like a timeframe to discharge, what would be in the most relevant at that point, so that they can focus their attention to that next step.” “I think setting up or educating the family on what is expected of them… what kind of tasks they need to learn as well as the timeline. And how to go about that. Because some families expect people just to come to them and teach. Whereas they have, it’s a give and take thing, you know, I’m here today, and I need to learn X, Y, and Z, or what do I need to learn.”

**Table 3:** Themes and illustrative quotations: suggested constructs for mobile health application development (path planning, information and support).

	N	Patient		Provider	
Domain/Theme		N	Illustrative Quotations	N	Illustrative Quotations
Domain 3: Path planning					
(a) Providing a discharge summary embedded in application	17	0		17	“[We should include] an updated discharge summary. A lot of families read the summary, and they are either confused by the jargon because it’s, you know, physician to physician or sometimes they don’t remember that that child had this or that, or this diagnosis that completely resolved etcetera, etcetera.”
(b) Embedded patient navigation assistance with parent navigators	21	21	“When my son had hearing loss, I don’t remember the name of the organization, but there was some organization that paired me up with a parent mentor that was super helpful. And I was just thinking, if there could be something like that [at our hospital]. Like my son’s primary need is in the pulmonary area. So if you could have like another parent of a kid with lung issues kind of partner up with you … I think that would’ve been massively helpful, and I mean, I’d totally be happy to do that with someone else. And explain all these different programs and insurance and how DNEs work and all that stuff. Like Regional and therapy. That was a lot to try and figure out, and took a long time to kind of navigate all of those. So I think something like that would be incredibly helpful, if there was like a volunteer system, you know, than they get pair you up with someone.”	0	
(c) Coordination with the medical home	22	7	“But it was quite a while before I even heard they had they had a medical home department. And I ended up working so much with our pulmonology care coordinator, nurse care coordinator. She’s done most of that stuff for me. But that would be super helpful to know, ‘cause I didn’t know they had that department for quite a while.”	15	“So, the, the medical home in [clinic] has sort of sheets on, like, okay, here’s your durable medical equipment, you know, until, so, sort of, putting it together, not, not just a, an electronic medical record but also one that’s parent-focused. So, it has everybody that I need to contact and, you know, the calendar and person, and what my insurance covers, and medications, and everything, just, we build that, it goes from our system to theirs and, and out to the community.” “And that’s what we see in the HRF [High Risk Infant Follow Up Program] environment… the breakdowns in that transition planning and the connections with the other services. So, sometimes they don’t have an appointment with pulmonology. They don’t have an appointment with cardiology, and then they’re, sort of, floating and then, that’s just increasing the anxiety around how do I care for my baby if I can get connected. And it is sort of, quarterbacking all that in the medical home. So, we, we often are failing primary care providers and walking them down to [clinic] because the community providers can’t manage some of the kids you guys are discharging. They can’t, they can’t connect them with the resources that can support them, so.” “Technically if they have a pediatrician, technically that pediatrician could be a medical home, if they’re, you know, supporting the family appropriately, right? Again it varies how how comfortable some of the community pediatricians feel connecting the family to, back to the specialist here, back to resources, when the, when there’s been those gaps. So that’s often an area that we are um intervening.”
(d) Facilitating appointment scheduling/referrals to specialists	29	9	“Sometimes I have personal things that I need to do. So, it’s a little hard trying to overlap that. They’re not, the therapies are not long it’s just they’re at the timing that like, most of the things that I need to take care of are the only available time. So, I try to make it as positive as I can.”	20	“And another part of the problem with accessing services and referrals outside is if we’re asking them to do so much, we’re asking them to make appointments with ten different specialists and call the Regional Center, and go to WIC, and call to get refills for your formula and your G-tube supplies, and they may, they may have 20 phone calls a month in that first two weeks to, to try to arrange all this in their life.”
Domain 4: Provision of information					
(a) Requested content area: accessing and using medications/durable medical equipment	99	79	“Going home … they teach you how to use everything, they show you how to do it. You have a good basic handle on how to use the machinery. But then it’s just using it by yourself, trying to make sure that you don’t do anything wrong that messes up and then end up sending your child back to the hospital. And if you accidentally miss this because you’re so worn out and tired, then you’re gonna mess up everything … it’s nerve-racking knowing that all of this is soon gonna be your, your issue, your things to deal with.” “feel like more than anything, like, the whole process of like, how the oxygen works … because obviously, they’re premature, so some babies de-sat … I feel like more of like, like, those, that kind of area, I would feel like it would be helpful if there would be more information.” “I mean I think all the equipment training was really helpful. Like the G-tube class was really helpful. But I had one nurse in particular who I remember really kind of like warned us how careful we needed to be and how easily he could end up back in the hospital. And most people didn’t really talk that way. So it was really helpful, some of her advice on how to keep your kid out of the hospital.”	20	“And this is, to build on that their medications and where they get them. And it’s an ongoing problem. Where, who refills my Albuterol? Who refills my Sildenafil? Because a pediatrician doesn’t refill all of those. And they could probably but sometimes, the CCS panel doctor and sometimes with the community pediatricians, they’re not going to touch the pulmonary meds, I don’t want you guys to do that, you check the electrolytes and you refill the sodium because I never give that. So, they don’t know where to get the refills.”
(b) Requested content area: community-based services	72	34	“And like she said, it’s hard, it’s hard. Like she said, her husband doesn’t even have a job. You know, and they just living and it’s hard. And a little help would do, like two times you could give us a food voucher, you know, when we go home, go here, here’s a, a gift certificate for some diapers, you know. Because I think it’s, it’s, it’s needed. You know? I’m, you know, I’m struggling.”	38	“I think you touched on the key point … when they do actually get the resources, it resonates with them. My last research was talking to these moms that were discharged. And the ones that said that the social workers offered it in the NICU, it helped them tremendously throughout … identifying them in the first place, to give them those resources are the hardest.” “Some of the units that I’ve been to, we do have weekly or monthly support group. And it’s family there discussing. And the topics may be different, and different things will come up, but, but things like doing the early intervention or regional center is really important, how do you get to it, how, what forms do you have to fill out, what are the shortcuts, you know. Those type of things can really come out in family support groups.”
(c) Requested content area: accessing mental health services	35	14	“I don’t remember being given anything. Unless I’m forgetting. I don’t think I was. I mean my OB would check in a tiny bit. I think I had one or two follow-ups with her. Like kind of just screening for post-, post-partum depression and things. I don’t think the NICU specifically did anything. But honestly, that would be super helpful. They could even just have like a counselor come and sit and offer their services, I would have loved that. Just, I like used to do that a little bit with a social worker and with some of the chaplains, and it was so helpful to have someone to talk to, but, yeah, I would love it if they had a counselor who was available to the parents. I wasn’t aware of it if they did.”	21	“The rates of postpartum depression that like we get from clinic are probably lower, that probably moms are maybe underreporting to the pediatrician. And then the big challenge then is linking them to community-based resources. One thing at least with the earlier postpartum moms that we see, they need to be depressed and need therapy, but they can’t get out of the house with the baby they don’t have a ride.”
Domain 5: Accessing support					
(a) Family support	34	27	“I just cheer her on. My mom, and my sister help me out a lot. We’re just like, cheering her on like, yes, you could do this. Like, she barely started walking. So, we just try to cheer her on in that, or we tell her oh, we’re going to go somewhere.” “I have support with my family. I will tell you that. I call my family for everything, okay, cause they’ve been there with kids. They have grown kids. So I try to basically call them and be like, hey, what do you do when this happens, or what happens when you do this? That’s what I do. I’m a crybaby to my family.” “I had a lot going on, you know, with my daughter and that really was my main focus and that really demanded all of my attention. But, you know, it wasn’t ‘til later that she was a lot stronger, and I could kind of sit there and relax a bit more. And then that’s kind of what I realized, you know, I am depressed. I do have a lot on my mind. I am almost at my breaking point emotionally, and it was just overwhelming and, you know, yeah, you can talk to your family, and you can talk to your spouse.” “Scared the hell outta me. But the person who watches him is his godmother. And she actually took G tube classes with me and everything like that. So she’s the only person that I can like legit trust to watch him and I don’t fear anything. And my job is in the same, you know, area, so I’m not too far away from him.”	7	“Social support is the same way … when I hear that ‘I’m living with my mother and my aunt helps,’ my stress level goes down because I know you have backups, and your other child can get cared for, and you can be here and if the dads not present, that’s a red flag.”
(b) Peer support	78	60	“You can talk to, you know, maybe other friends if you are to have children who may or may not have their issues and whatnot. But just somebody to listen, somebody you can just be privy to, somebody. I mean honestly even if it were Joe Schmo on the other end of an email, you know, just somebody you can even type a long 30-page novel to about how you feel and what you’re going through and how all that makes you feel and how your world really does feel like it got flipped upside down and inside out.” “She was saying, at first of course you’re scared because now you’re coming home with a baby that has a disability and you don’t know how you’re going to cope, and try to go with your everyday life too, but, it was a great experience because, they had already gone home. Unfortunately, their son had to come back to the hospital, but, it was a great learning experience for us because, like, that’s okay. This is what we did wrong. This is what you shouldn’t do wrong. So all the pointers that they gave us were very good.”	18	“Pairing somebody who’s gone through the process and knows what it takes, so maybe knows the next step like would be ideal a situation, like a mommy support group, you know, parent support group.”
(c) Religious support	10	10	“I don’t know if he was like a chaplain or a reverend, or I’m not sure. But, he came and kind of, you know, talked to me a little bit and told me about the prayer center.” “My family … But I also found, a lot of support through the church.”	0	
(d) Support group	21	10	“To be honest with you, people are all different. A section for both would be great. Like, if they wanted to go find a group that they can talk in, or if they were more, like me, not so apt to just finding a whole group of people you don’t know to spill your business in front of, and you just want to talk to like, one person, simple, just, straight to the point, having somebody do Facetime like this or just chatting with them would, would help a lot, an unbiased opinion to talk to, right then and there.” “Yes and then also maybe having like a little chat group with other parents and just talk about, you know, just everything that they went through, and just so I won’t feel alone. ‘Cause um sometimes like it’s really hard for me to talk to other people about what I went through because it’s just really traumatic for me.”	11	“I highlight what [other focus group participant] was saying about support groups because I also listed it as one of the things that we don’t have and would really be important for us to have because simply getting over the language barrier wouldn’t be enough I don’t think. The cultural barrier and having a support group that you know is non-judgmental—the support group would explain to you, this is my trick for having two kids and bringing them both to the appointment with my other kid to a therapy. And this is how I get around it. That support group might explain, this is the train I take, these are the days I make this appointment, this is when doctor so and so is in.” “Some of the units that I’ve been to, we do have weekly or monthly support groups. And it’s family there discussing. And the topics may be different, and different things will come up, but, things like doing the early intervention or regional center is really important, how do you get to it, what forms do you have to fill out, what are the shortcuts, you know. Those type of things can really come out in family support groups. So you’re gonna have family who’s been here longer period of time, have seen more things going on, and parents that are just here for like one week and still kind of wide-eyed and learning all of the resources that’s available to them.”

**Table 4:** Themes and illustrative quotations: suggested constructs that a mobile health application might improve.

	N	Patient		Provider	
Domain/Theme		N	Illustrative Quotations	N	Illustrative Quotations
Domain 6: Engagement with providers					
(a) Communication with pediatricians	34	6	“And, you know, I think a lot of it too was, again, feeling that she looked and seemed so healthy … And I felt for a long time that somehow I was being inadvertently blamed for her not gaining more quickly. Nobody told me about that. You know, when we are, they do the transition from being in the hospital to going home. But we basically even now, we still have, you know, pediatrician’s appointments and whatnot and I have to keep a food diary and, you know, it’s daunting, it’s just, it’s constant. I didn’t get that memo, apparently.”	28	“There’s a lot that we can do for that that we don’t …. I called your pediatrician last week, we’ve talked, they’ve asked a bunch of questions, I’m going to let them know, the day before, again, that you’re going home, and this is the final word, and if they have any more quest-, that would make them feel more confident if they had some of them don’t even know that we call their pediatrician at all, even if we have.” “Yeah. I know when I ask the front-line providers to call the pediatrician with an update, call them with a handoff and all that, there’s many times that I get pushback and say, well, give the parents the discharge summary, they’ll plan it out. And that really needs to be enforced from not just me, but from somebody higher up than me to call, even just, maybe not the simple ones, maybe have some guidelines. But I know on, on the complex kids, I’ll often call and deal them out. You know, this is baby with X, Y, Z, can you, do you have the resources to take of it in your clinic? sometimes they’ll say no, I’m the only pediatrician, I’m only here once a week and it’s nurse practitioners.”
(b) Communication and importance of clinical care coordinators	21	9	“I was thinking something that could be helpful at discharge is maybe giving you the contact info of like your nurse care coordinator or someone from the high-risk clinic. Those people have been amazingly helpful to me, but I didn’t have their contact info at the beginning, and I didn’t realize how much they could help me … They’ve advocated for me. They’ve told me what I can push for. ‘Cause I think at the beginning I didn’t understand how much you had to advocate for your kid, so I would be told no by insurance and didn’t realize I kind of just had to keep pushing and to get stuff. And so those people have done that for me.”	12	“Cause I literally did spend two hours trying to get to these clinics and get them numbers, and the dates and times.” “Yeah. I think the fact of the matter with regional center … we work with them continuously with all of our patients, is that advocating for your baby is number one. They are very impacted. They have too many patients, not enough therapists … so the ball often gets dropped. And I think part of the education piece should be you need to continually call, they are not going to call you.”
Domain 7: Barriers to care after discharge					
(a) Health literacy	11	5	“And it would’ve been so helpful, though, too, to understand more what tests were being done and for what reasons and what the results mean. And, okay, she had a blood gas number of X. Okay, well what does that mean? it’d be nice to have some sort of way to like decode all of this stuff. So, it’d be great if, you know, that was the case.”	6	“I think you know there’s a lot that … their baseline reading level may not be high, so the info we’re throwing is um really difficult to comprehend.”
(b) Language/cultural barriers	13	0		13	“I feel like sometimes there’s a language barrier because I feel families might not be familiar with all the different resources that are available, they might be scared that they don’t speak their language, that they won’t get the support that other families need.” “I highlight what [co participant} was saying about support groups because simply getting over the language barrier wouldn’t be enough I don’t think. The cultural barrier and having a support group that you know is non-judgmental um, that support group would explain to you, this is my trick for having two kids and bringing them both to the appointment with my other kid to a therapy. And this is what I, this is how I get around it.”
(c) Transportation/housing	21	14	“You know, my biggest, my biggest thing is maybe … I know it’s probably far-fetched, but maybe you guys could have some transportation for people that really need [it] … Maybe people on the bus or, or walking, or that don’t have transportation, maybe we could have maybe a free shuttle.”	7	“And then, some of these parents don’t have, like, a car for example, and so, it’s public transportation to all the different, like, offices and Regional Centers and some specialist’s appointments can be also really hard for them too?”
Domain 8: Parenting role and confidence					
(a) Parenting confidence and parenting role	20	14	“I feel like the only thing that I do remember one of the nurses telling me that, she, that to not really like be scared but more like to see like, it’s really hard to explain because more than anything, I was just scared because I had never seen a premature baby.” “It’s for regular babies, that’s why I feel like it would be more difficult because, there was some moms that I could, like, relate to. Most of them never really had the experience of prematurity. So, I feel like that would be useful to like, have something more specifically to premature baby.”	6	“Parenting confidence is ironic in that first-time parents who deliver in a birthing center, take their kids home on day two will tell I do not know what I’m doing. It is the same lack of confidence that, that families in the NICU feel except for the fact that this child is more likely to have a big setback with their lack of confidence. And that is what drives the fear and the anxiety. Not that more so, that they don’t know what they’re doing like any other parent, but if another parent forgets to feed their kid they’ll just catch up on the next one, and in this one, this feeding was supposed to go over a pump, over two hours, this child has some insulin instability, glucose instability, and that, anxiety is definitely a big deal.” “Yeah. I think that, I don’t know, just going to what [participant] was saying which is about parents being at the bedside and just forming those bonds … I think that kind of fosters a lot awareness of the baby and builds their confidence that they know that they can provide for their baby, even if they’re not a doctor and they’re not a nurse, and they’re not a specialist, like, they can, they have the means to be able to provide for their baby and comfort their baby.”

## Data Availability

The data presented in this study are available on request from the corresponding author.

## Appendix A

Table A1: Interview Guide.

**Table A1:** Focus group interview guide.

Construct 1: Information	The first set of questions are about how you would like information presented to you on a web based application. (1) How do you think you would feel more empowered to understand your child’s medical situation? (2) How would you like information presented to you? (3) What do you think about educational videos? Would the information stick better if it was a story (a narrative) rather than just information? (4) What kind of information would be useful to you? Printed materials? (5) Would communication via your smartphone be useful? What kind of information would be useful?
Construct 2: Social Supports	Would you be interested in having a social support system via the web based platform? (1) How important do you think this is for you? (2) Why would it be important? (Probes: Mental health, your feelings, your care of your baby) (3) Do you think it would be easier to learn about taking care of your baby if you could be in a group with other families? (4) Do you think having group visits for part of your visit to the High Risk Infant Follow Up clinic would be helpful? (5) What kinds of applications (Facebook, Instagram, etc.) do you use?
Construct 3: Community based services	How would a web based platform help you access community based services like: (1) Regional Center (2) Supplemental Security Income (3) Public assistance programs like WIC (Food stamps), SNAP? (4) How would it be useful for you to know more about them? (5) How about your pediatrician, medical visits and the medical home? (6) What could we do to make the information more approachable to you
Construct 4: Mental Health	The next set of questions are about how you feel, your mental health-do you think a web based platform might be useful to you as a resource? (1) Do you feel like you could talk to anyone about your feelings? (2) What could we do to make that process easier? (3) Do you know about mental health services?
